# The origin of color categories

**DOI:** 10.1073/pnas.2400273121

**Published:** 2024-12-30

**Authors:** Daniel J. Garside, Audrey L. Y. Chang, Hannah M. Selwyn, Bevil R. Conway

**Affiliations:** ^a^Section on Perception, Cognition, Action, Laboratory of Sensorimotor Research, National Eye Institute, NIH, Bethesda, MD 20892; ^b^National Institute of Mental Health, Bethesda, MD 20892

**Keywords:** categorization, language, color

## Abstract

To what extent does concept formation require language? Here, we exploit color to address this question and ask whether macaque monkeys have color concepts evident as categories. Macaques have similar cone photoreceptors and central visual circuits to humans, yet they lack language. Whether Old World monkeys such as macaques have consensus color categories is unresolved, but if they do, then language cannot be required. If macaques do not have color categories, then color categories in humans are unlikely to derive from innate properties of visual encoding and likely to depend on cognitive abilities such as language that differ between monkeys and humans. We tested macaques by adapting a match-to-sample paradigm used in humans to uncover color categories from errors in matches, and we analyzed the data using computational simulations that assess the possibility of unrecognized distortions in the perceptual uniformity of color space. The results provide evidence that humans have consensus cognitive color categories and macaques do not. One animal showed evidence for a private color category, demonstrating that monkeys have the capacity to form color categories even if they do not form consensus color categories. Taken together, the results imply that consensus color categories in humans, for which there is ample evidence, must depend upon language or other cognitive abilities.

## Significance

A hallmark of intelligence is the use of concepts. Are people innately equipped with concepts? Prior research has addressed the question using color, because color is experienced categorically: color categories reflect concepts of color. This study tested for color categories in macaque monkeys, a species with the same visual-encoding systems as humans. If color categories are innate products of vision, monkeys should have them. The data were analyzed with sensitive computational models, which showed that monkeys do not have consensus color categories, unlike humans. One monkey had a private color category, suggesting that the capacity to form color categories is innate. The results imply that cognitive mechanisms such as language are required for the expression of consensus color categories.

Color categories are identified by color terms, of which the Basic Color Terms are considered prominent (1). One hypothesis is that a subset of these terms—red, green, blue, yellow—express universal concepts (2, 3) that are endowed by hard-wired neural mechanisms present at birth (2, 4–6). This idea, put forth 150 y ago (7), predicts common patterns in color naming across cultures (8–10) and finds some neurophysiological support in human infants (11, 12). Behavioral work in infants also provides evidence for a biological origin of color categories (13, 14) and suggests that color categories may build on an innate scaffolding defined by retinal cone-opponent mechanisms (15). But the infants in all these experiments were at least several months old, by which stage they would have had substantial cultural exposure, so the evidence that they express color categories cannot be conclusively ascribed to an innate origin. Another hypothesis is that color categories emerge in development, instructed by language and culture (16, 17), possibly involving an interplay of innate and developmental factors (18–22). Support for this hypothesis is provided by the variability in color-naming patterns across languages (16–19, 22, 23). The notion that at least some aspect of color category behavior is acquired through experience is consistent with computational theories of color term evolution that do not require innate mechanisms (23–26). But the extent to which color categories are innate remains unresolved (27–29) (see also chapter 6 in ref. 30).

Another approach to studying the origin of color categories could be provided by behavioral experiments in trichromatic nonhuman primates (29, 31–33). These animals lack language but have almost identical perceptual systems to humans, and they presumably have the same perceptual color space (34–37) and similar mechanisms of visual cognition (memory and decision-making) (e.g., refs. 37 and 38). The few studies following this approach have come to different conclusions: one found color categories in macaque monkeys consistent with categories in human adults (31), implying that color categories do not depend on language and may be innate. This study inadvertently made comparisons substantially easier for cross-category than within-category trials, undermining its conclusion (29). A later study tested for the existence of color categories across a limited range of colors and found a blue-green boundary in humans but not baboons (29, 39). The lack of color categories in the baboons might reflect the limited survey of color space [blues and greens are categorized with high variability in all languages, including English (23)]. A third study, designed to investigate visual working memory, uncovered a different pattern of results in the two animals tested (38), raising the possibility that the two animals had private color categories.

## Measuring Color Categories in Macaque Monkeys.

**Fig. 1.**
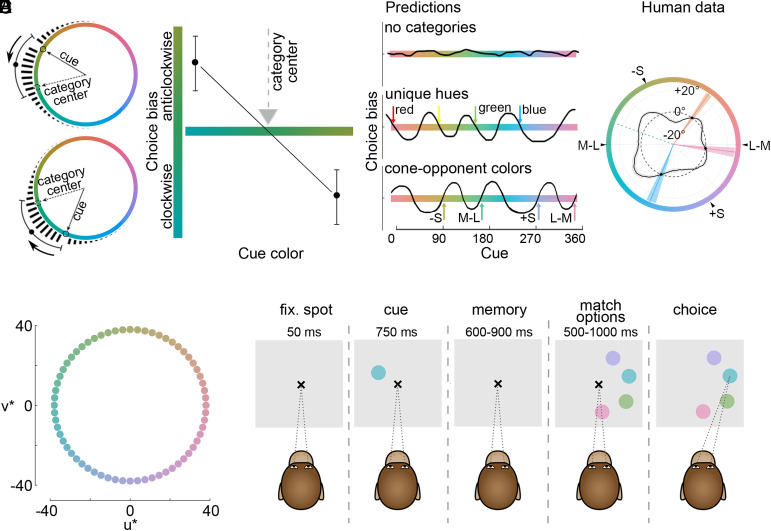
Nonverbal paradigm to recover color categories in nonhuman primates. (*A*) Predicted distribution of choices for two cues if a color category exists at the specified location in the color space (dashed arrow). The average of the distribution of choices will be biased counterclockwise from the cue if the cue is displaced clockwise to the category center (*Top*) and biased clockwise from the cue if the cue is displaced counterclockwise to the category center (*Bottom*). This pattern of results would be captured as the zero-crossing of the negative slope in a plot of the choice bias versus cue color (*Right*). (*B*) Predicted pattern of results for three hypotheses: no categories (*Top*); categories defined by attractors to the four common basic color categories (*Middle*); and categories defined by repellers to the cone-opponent retinal encoding mechanisms (*Bottom*). (*C*) Data obtained in prior work on a related task in human subjects showing evidence of four color categories. Though there are only three zero-crossings in the dataset presented here [that of Bae et al. (43)] the data shows a periodic structure with four cycles, with a fourth attractor point suggested at roughly 180° (green). See *SI Appendix*, Fig. S2 for two additional datasets in human subjects including the prior work of Panichello et al. (38) and data using the present task; all infer the existence of four consensus color categories. The negative slopes demarking category centers are recovered by tracing the line in a counterclockwise direction, at points where the trace crosses the dashed circle marking zero choice bias. For reference, arrowheads show the colors that would isolate the retinal cone-opponent encoding mechanisms (L − M, M − L, +S, −S; where L, M, S are the three cone types). (*D*) Sixty-four colors defined in CIELUV color space. (*E*) Animals were trained to initiate a trial by fixating a small cross on a computer monitor and to maintain fixation throughout the trial until the fixation cross disappeared, which was their instruction to make a choice; trials in which the animals broke fixation were aborted. A 3° diameter cue was presented within the central 2.5 to 6°, followed by a variable memory delay (600 to 900 ms) and the presentation of four choice options. To mitigate impulsive choices, the choice options were shown for a variable amount of time (500 to 1,000 ms) during which the animals needed to maintain fixation to avoid aborting the trial. After the fixation cross disappeared, the animals were free to make their selection.

Addressing the question of color categories in monkeys requires overcoming several challenges. First, how can color categories be measured nonverbally, without teaching the animals the categories or reinforcing idiosyncratic biases (40, 41)? Second, how should the color stimuli be chosen (33)? For example, choosing colors according to their wavelength equivalent (31) is not appropriate (29). Third, how can data across the full circle of hues be obtained so as to avoid missing categories (39)? A match-to-sample paradigm, using colors defined in a nominally uniform color space (42) (Fig. 1), provides a potential solution to these challenges, because, as demonstrated for human subjects, color categories introduce biases in the distribution of matches (43). So color categories could be inferred from any observed biases across the space of colors without requiring participants to understand or produce linguistic labels. The logic of nonlexical assays of color categories stems from cross-cultural studies of the Dani people (2) and subsequent challenges to it (44).

In the standard paradigm used in many experiments on working memory and adopted to infer color categories, the subject is asked to match the color of a cue to a continuous ring of colors (Fig. 1*A*). This works in human participants who can follow instructions but introduces complications in monkeys because it is not clear how to reward the animals (animals must be rewarded to motivate them to do the task). Rewarding the animals for making incorrect responses could reinforce idiosyncratic biases; meanwhile, rewarding the animals for only correct responses is impractical: the response metric (an eye movement or pointed finger) is likely confounded by motor noise, yielding too few trials recorded as correct for the animal to receive sufficient motivation. To overcome these complications, we adapted the paradigm using discrete colors (Fig. 1*D*) and an alternative-forced-choice task in which a direct match to the cue was available in every trial and the monkeys were only rewarded for making the direct match with an eye movement (Fig. 1*E*). The precision of the eye tracker (∼0.3° of visual angle) was considerably finer than the size of the choice options (3° diameter). One consequence of the adapted paradigm is that it requires a much larger number of trials to satisfactorily assess the similarity relationships of each color to every other color. Four animals performed the task, completing a total of 209,456 trials over 232 sessions (*SI Appendix*, Fig. S1).

We followed the logic of ref. 43, which rests on the assumption that underlying categories cause biases in matching: if a subject has a color category, the category center will be an attractor in the color space. An attractor would be captured by a zero-crossing of negative slope in a plot of choice bias (Fig. 1*A*). Repellers, meanwhile, would be captured by zero-crossings of positive slope. The approach is data-driven, so it will recover whatever categories exist; nonetheless, before collecting the data, we considered three possibilities. First, that the monkeys would show no color categories, predicted by the work sampling a limited range of colors in baboons (29) (Fig. 1 *B*, *Top*). Second, that the monkeys would show four color categories, predicted by attractor points defined by the four putatively universal basic color terms (Fig. 1*B*, *Middle*). Third, that the monkeys would show four color categories, but predicted by data in human infants corresponding to repeller points defined by physiological cone-opponent mechanisms not basic color terms (15) (Fig. 1 *B*, *Bottom*).

For reference, Fig. 1 *C* shows the results of the prior study in human adults (43), where the results are most compatible with the second or third possibilities outlined above. Note that the authors of the original study show that the data is best explained by a model with four attractors, even though there are only three zero-crossings [this result has been replicated by others (38)].

The four recovered color categories do not perfectly line up with the four common basic terms; instead, they correspond to pink, orange, green, blue. The discrepancy may arise because of limitations imposed by ensuring equal saturation and luminance among the colors (the resulting set of stimuli has neither a good red nor a good yellow) (see ref. 43). Alternatively, the recovered categories may correspond to repeller points (positive zero-crossing) defined by the poles of the cone-opponent mechanisms (arrowheads, Fig. 1*E*), with the exception of the L − M pole. Yet another possibility, not mutually exclusive, is that the color categories recovered in any task depend on both the utility of the color category [which varies among categories (6, 23, 44)] and the nature of the task. This latter explanation is favored by the observation that the task developed by Bae et al. (43) recovers a fewer number of consensus color categories than the typical eight consensus (basic) color categories in English.

The recovery of four categories has been replicated in English speakers (38) including by us (*SI Appendix*, Fig. S2). The four categories are at roughly matching locations across studies (*SI Appendix*, Fig. S2).

**Fig. 2.**
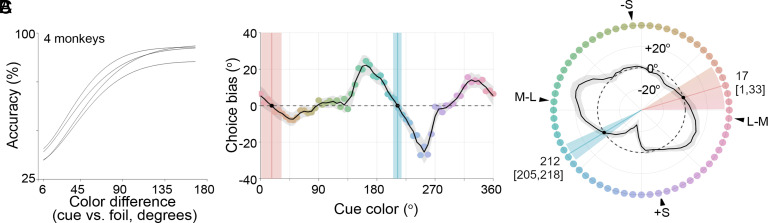
Macaque monkeys appear to show two consensus color categories when the data are analyzed with a mixture model that computes the average distribution of the choices for each cue. (*A*) Psychometric functions for the four animals showing the accuracy of the matches as a function of the difference in hue angle between the cue and the foil that is closest in color to the cue. The easiest trials were defined as those where the foil color nearest to the cue color was almost on the opposite side of the color circle. See *SI Appendix*, Fig. S1 for 95% CI. (*B*) Mixture model results averaged across the four animals. The data were subsampled so that the same number of completed trials for each animal (24,526) were included in the analysis, corresponding to [9,475, 9,694, 10,665, 9,783] incorrect trials respectively for PO, CA, BU, MO. Error shading shows 95 %CI. The data recover two significant negative-slope zero-crossings (black dots), corresponding to two color categories. (*C*) Polar plot of the results in (*B*) with the zero-crossings of the negative slope (following the trace counterclockwise) again indicated by black dots. The angles of the two inferred color categories [95 % C.I.] are 17 [1, 33] (a peach color) and 212 [205, 218] (a teal color).

The four animals performed well on the task, achieving lapse rates of 7.0%, 7.1%, 5.8%, and 14.3%, on the easiest trials (Fig. 2*A*; the plots of individual animals showing 95% CI are provided in *SI Appendix*, Fig. S1). The results averaged across the four animals, analyzed using the mixture model as used previously to analyze data collected in humans (43, 45) (*Methods*), provided clear evidence of choice biases (Fig. 2 *B* and *C* and *SI Appendix*, Fig. S4). But the apparent color categories shared among all animals inferred using this analysis do not support any of the predictions: the animals appeared to show two consensus color categories, not four as in humans. To the extent that the macaque is an accurate model of a nonlinguistic human, these results show that the four color categories manifest in humans are not innate. The centers of the two apparent consensus color categories recovered in the monkeys were at 17° (a peach color) and 212° (a teal color). All four animals showed evidence consistent with the pattern observed in the average data (*SI Appendix*, Fig. S5).

## Two Possible Explanations for Choice Biases in Macaque Monkeys.

**Fig. 3.**
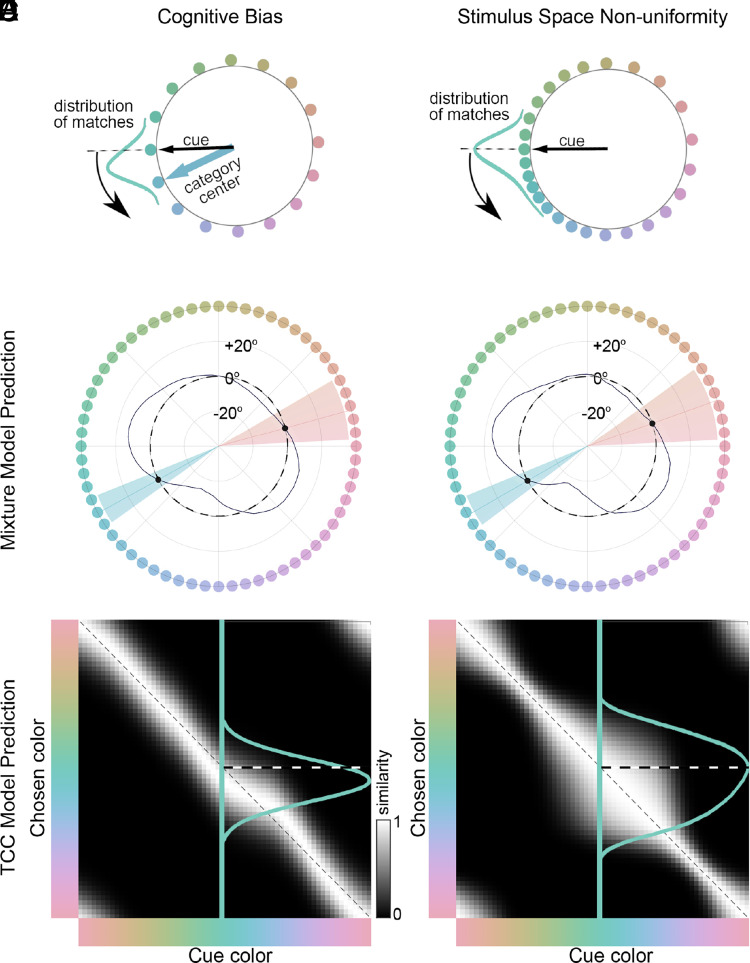
Computational simulations showing that color choice biases recovered by mixture model (43, 45) could be explained by a nonuniformity in the stimulus space, without invoking cognitive mechanisms. (*A*) Color matches made by an agent with a cognitive color category, using a paradigm with stimuli that uniformly sample a truly uniform perceptual color space (gray circle). The distribution of matches has a peak biased toward the category center. (*B*) Color matches made by an agent lacking a cognitive color category, with stimuli that nonuniformly sample an underlying uniform color space (gray circle). The distribution of matches is biased toward the densely sampled region of the space because there are more choice options. The average of the distribution of matches is similarly biased counterclockwise to the cue in both a and b, although the similarity functions differ in shape. (*C*) Mixture-model analysis of a simulated dataset with a cognitive bias; Code for F3c (*D*) Mixture-model analysis of a simulated dataset with a stimulus space nonuniformity. Code for F3d (*E*) Similarity matrix for the simulated data analyzed in *C*; axes are CIELUV color space. Each column shows the similarity function for the corresponding cue on the *x* axis. The trace shows the similarity function for the cue in *A*. Note that the shape of the similarity function is symmetric like the distribution of matches. Code for F3e (*F*) Similarity matrix for the simulated data analyzed in panel *D*; other details as in *E*. Code for F3f. The shape of the similarity function is different in *E* and *F*, yet both have the same mean bias relative to the cue. The similarity function in *F* and the distribution of matches in *B* are both asymmetric but differ in shape because the color spaces are different in *B* and *F*.

The colors we used were sampled in CIELUV—a colorspace defined by the International Commission on Illumination with the goal of being perceptually uniform. But it has long been recognized that there may be nonuniformities in all color spaces, including this one (42); some authors have argued that perceptual uniformity may be task-dependent or simply unattainable (46, 47). One might even suppose that if language influences color perception, as stipulated by the cultural-relativity hypothesis (17), then all color spaces generated by human observers could be shaped by language. Could the pattern of results in Fig. 2 *B* and *C* be attributed not to a true cognitive category (Fig. 3*A*) but to unrecognized distortions in the presumed uniform space of colors (Fig. 3*B*)? Both explanations could introduce biases in the distribution of matches (the mean for the distribution of matches will be biased away from the cue, curved arrows Fig. 3 *A* and *B*).

The difference in the two explanations can be understood by considering the relationship between two neighboring colors in the color space. For the cognitive-bias account, there is an asymmetry between neighbors if there is a category center nearby. The color further from the category center will be more likely mistaken for the color closer to the category center than the other way around. In the nonuniform color space account, however, there is no asymmetry in mismatches between neighboring colors. Both explanations lead to a distribution of matches that is shifted away from the cue. We refer to the distributions of matches for a given cue, plotted in CIELUV, as the similarity function for that cue, and the family of similarity functions for all cues as the similarity matrix. A cognitive bias would yield similarity functions of constant width for all cues around the color circle, but the function peaks would deviate from their respective cues depending on the proximity of the category center (Fig. 3*A*). A stimulus space nonuniformity, meanwhile, would yield an asymmetric similarity function which peaks at the cue (Fig. 3*B*), and it will vary in width and shape depending on sampling density of the true underlying uniform color space, with regions that are relatively densely sampled having broader distributions.

To illustrate that the results of a mixture-model analysis could be explained by either a cognitive-bias account or a stimulus-space nonuniformity, we generated two sets of simulated data. One dataset was generated with a simulation that used a uniform space and a bias arising from two cognitive categories, and the other dataset was generated with a simulation that used nonuniform sampling of an underlying uniform color space and no cognitive categories. The datasets from both simulations gave rise to the same pattern of results when analyzed with a mixture model (Fig. 3 *C* and *D*). These simulated datasets were specified to match the pattern of biases recovered in our behavioral results (compare the simulated results in Figs. 2*C* with 3 *C* and *D*).

To decide which of these explanations best accounts for the macaque behavioral data, we developed computational models of each mechanism and analyzed the data with an extension of the target confusability competition (TCC) model (48) (*Methods*). The standard implementation of the TCC model assumes the same similarity function for each color. The version developed presently allows the shape and/or location of the similarity function to vary per color (we refer to the modified TCC model as TCC-v, for “vary”). Let us consider three versions of the TCC-v model. First, the simplest (“null”) model has the same similarity function for each color, centered on the target color (this is conceptually equivalent to the original TCC model). This model has two free parameters (*σ*, $d^{\prime }$) which control the width of the Gaussian similarity function and the amount of noise present in the system. Second, the “cognitive bias” version of the model specifies similarity functions for each color with peaks that can deviate from the target color but in which all similarity functions for the set of colors have the same symmetric (Gaussian) shape (Fig. 3*A*). In addition to *σ* and $d^{\prime }$, this model has an additional set of 64 parameters which control the offset of the peak of the similarity function—one for each color. The result is a similarity matrix that has a band of constant height along the inverse diagonal but deviates to one side near color-category locations in the color space (Fig. 3*E*). Third, the “stimulus-space nonuniformity” version of the model has a fixed similarity function across colors, but the perceptual distances between colors can differ, which results in a similarity function which is distorted when it is plotted as a function of stimulus index. In addition to *σ* and $d^{\prime }$, this model has an additional set of 64 parameters which control the relative angular perceptual distance between colors. This results in a similarity matrix that is symmetric about the inverse diagonal, but which bulges out away from the diagonal at locations in the true underlying color space that are oversampled by CIELUV (Fig. 3*F*). The stimulus-space nonuniformity model and the cognitive-bias model have the same number of parameters.

To recap, the similarity matrix in Fig. 3*E* is constructed using the same data as the polar plot in Fig. 3*C*, and the similarity matrix in 3*F* is constructed using the same data as the polar plot in Fig. 3*D*. Panels *C* and *D* use the standard mixture-model approach which does not allow an asymmetry in the shapes of the similarity functions. Panels *E* and *F* use the TCC-v approach, which permits the similarity functions to vary. The results of these computational simulations show that different underlying mechanisms could explain the appearance of choice biases in color-matching tasks when evaluated with the standard mixture model.

**Fig. 4.**
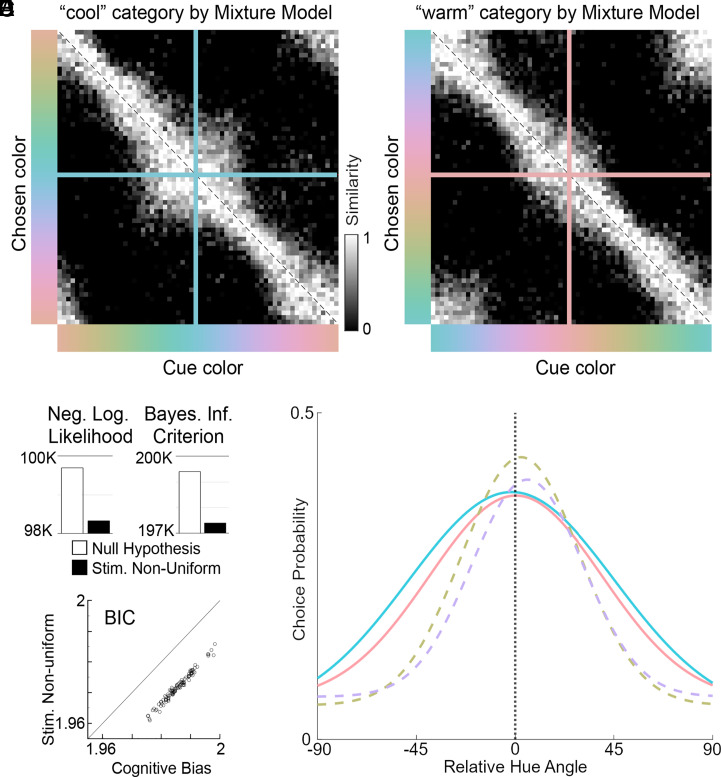
Similarity matrices for behavioral data averaged across four monkeys. (*A*) Data centered on the teal-colored category recovered in the mixture model. (*B*) Data centered on the peach-colored category recovered in the mixture model. The pattern of results in *A* and *B* is better predicted by stimulus space nonuniformity (Fig. 3*F*) than cognitive bias (Fig. 3*E*). (*C*) Negative Log Likelihood (*Left*) and Bayesian Inference Criterion (BIC, *Right*) of the fit of the null model and the stimulus-space nonuniformity model. (*D*) BIC values of the fit provided by the stimulus-space nonuniformity model were always lower than BIC values of the fit for the cognitive bias model for 100 bootstrap repeats of the analysis. For each bootstrap repeat, the number of trials for each animal were the same, set by the animal that completed the smallest total number of completed trials, and that number of trials was drawn with replacement from the total number of completed trials for each animal. The stimulus space nonuniformity model and the cognitive bias model have the same number of parameters. (*E*) Gaussian fits (extracted from a mixture model) showing broader gaussians at the two attractor points (solid lines) than at cues 90° offset (dashed lines).

In an additional version of the TCC-v model (“free-similarity”) we treat every cell in the similarity matrix as an independent model parameter (Fig. 4 *A* and *B*; the data for both panels are the same, centered on one or the other of the putative category centers recovered in the mixture model; free similarity matrices for the individual animals are shown in *SI Appendix*, Fig. S6). The free-similarity matrix makes no assumptions about the underlying functions that determine the similarity between any pair of stimuli (in contrast to the prior models which assume a Gaussian with specific manipulations). Such a model is very flexible, allowing nonhypothesized mechanisms to surface (For example, a “favorite color” bias would appear as a vertical line.). We can ask: is the average free similarity matrix better explained by the stimulus space nonuniformity model or the cognitive bias model? In other words, do the panels in Fig. 4 *A* and *B* show mirror symmetry with bulges about the diagonal (like Fig. 3*F*) or asymmetry about the diagonal without bulges (like Fig. 3*E*)?

By visual inspection, the data are better explained by the stimulus space nonuniformity model, a conclusion affirmed by statistical tests (AIC/BIC for null, cognitive bias, and stimulus-space nonuniformity models respectively are [199,397, 198,005, 196,792]/[199,416, 198,616, 197,402]). First, the stimulus space nonuniformity model provides a better fit of the data than the null model (Fig. 4*C*). Second, the data are better explained by the stimulus-space nonuniformity model than the cognitive bias model (Fig. 4*D*). These results show that the apparent two consensus color categories recovered by the mixture model are explained by a nonuniformity in the underlying color space and that there is a nonlinear mapping of a true perceptually uniform color space and the presumed uniform color space we used. These results therefore suggest that macaque monkeys do not have any innate consensus color categories. If the macaque is an accurate model of the human, then the results imply that humans do not have innate consensus color categories either.

**Fig. 5.**
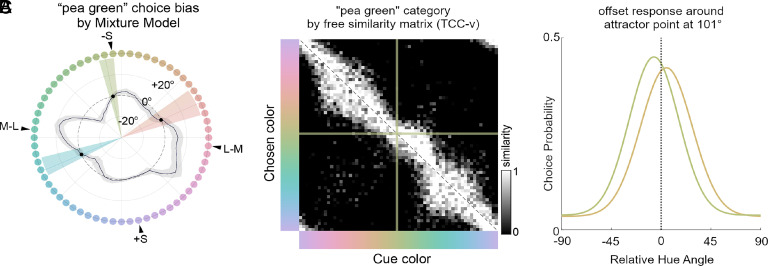
Color-matching data for one monkey (CA) showing evidence for a cognitive color category bias. (*A*) Mixture-model analysis (same format as Fig. 2*C*). (*B*) Free-similarity matrix (same format as Fig. 4 *A* and *B*) with an asymmetry in the green region indicated by the cross. (*C*) Gaussians extracted from the mixture model on either side of the identified attractor point (101°) showing offsets. Plotted curves are for hue angles 90° (*Right*, offset to positive values) and 112.5° (*Left*, offset to negative values).

The data presented so far are for the four animals combined, which allowed us to assess the existence of consensus color categories. But we noted that the individual animals showed some idiosyncratic patterns of results (*SI Appendix*, Figs. S5 and S6). By mixture-model analysis, one animal showed not only the two consensus choice biases that are explained by stimulus space nonuniformities but also a third bias, for pea green (Fig. 5*A*). The free similarity matrix for the data from this animal shows an asymmetry about the diagonal at this location in the color space (Fig. 5*B*), showing that this animal has a cognitive bias—a true color category—for pea-green, in addition to the consensus biases driven by stimulus-space nonuniformities. The result in this animal, and trends that the other animals also have idiosyncratic cognitive biases, show that macaque monkeys have the potential to form cognitive color biases, and that the TCC-v model can recover them. This is important not simply because it confirms that the ability to form cognitive categories does not require language, as suggested previously (38), but also because it shows that if macaques had consensus color categories, we would have been able to observe them. AIC/BIC for null, cognitive bias, and stimulus-space nonuniformity models, fit to just this animal’s data, are [97,181, 96,352, 95,242]/[97,200, 96,962, 95,853], respectively.

## Revisiting the Human Data.

**Fig. 6.**
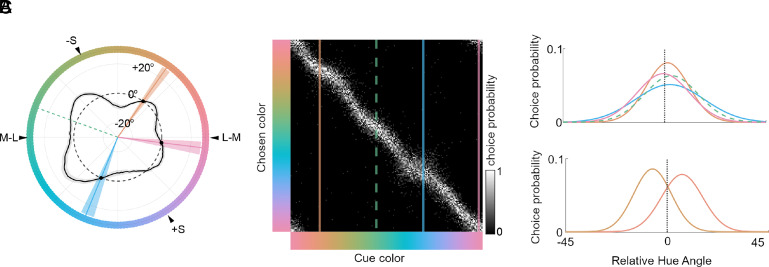
Reanalysis of the data of Bae et al. (43)—what mechanisms underlie the observed biases? (*A*) Mixture model analysis of the data of Bae et al. (43) (as in Fig. 1*C*, reproduced for easy reference). (*B*) Choice probability matrix (*SI Appendix*, Fig. S9 of the same data, with the attractor points recovered by the mixture modeling analysis highlighted. (*C*) Gaussian fits extracted from the mixture model. *Above*: Gaussians at the attractor points, showing a range of widths. *Below*: Gaussians at either side (*Right*, 38° and *Left*, 70°) of the orange attractor (54°), showing offsets toward the attractor.

The analysis of the average data collected in monkeys shows no evidence for consensus cognitive biases and strong evidence for stimulus-space nonuniformities, which suggest there are no consensus color categories in monkeys, in contrast with humans. But this conclusion depends on 1) the capacity of the task and analysis to recover cognitive biases; and 2) the existence of consensus cognitive color categories in humans. Regarding (1), the data in one individual monkey show clear evidence for an idiosyncratic (private) cognitive bias (Fig. 5), so we are reassured that the task and the analysis can recover cognitive biases should they exist. Regarding (2), we used the TCC-v model to reanalyze three datasets collected in human subjects by three independent groups (Fig. 6 and *SI Appendix*, Fig. S2). The analysis recovers evidence for both stimulus space nonuniformities and cognitive biases: the choice probability matrix (Fig. 6*B* and *SI Appendix*, Fig. S9) shows deviations from the diagonal, like Fig. 3*E*, as well as dilations along the diagonal, like Fig. 3*F*. For example, the blue attractor and the pink attractor are associated with relatively wide distributions of matches (Fig. 6 *C*, *Upper*), indicative of stimulus-space nonuniformities, while the orange attractor is associated with a relatively narrow and asymmetric distribution of matches (Fig. 6 *C*, *Bottom*), indicative of a cognitive bias. The attractors associated with stimulus-space nonuniformities also show an asymmetry about the inverse diagonal which is indicative of a cognitive color category.

For quantitative assessment, we fit generative models to these data. For a null model, a cognitive bias model, and a stimulus-space nonuniformity model we find AIC values of [76,563, 74,864, 75,585]. These results imply that the cognitive and stimulus-space nonuniformity models both explain the data better than the null model; of the two non-null models, the cognitive model has greater explanatory power. The relative advantage of the cognitive-bias model is also evident from the BIC values ([76,577, 76,191, 76,912]). We interpret the human data as evidence that both stimulus-space nonuniformities and cognitive biases interact to underlie the apparent consensus color categories evident in the mixture-model results. In other words, we do not believe that some subset of the apparent consensus color categories observed in humans can be entirely discounted as caused by stimulus-space nonuniformities, unlike the apparent consensus color categories in monkeys, which appear to be entirely attributed to stimulus-space nonuniformities.

## The Potential Impact of Task Differences.

We modified the continuous matching task to make it suitable for use in monkeys. The two modifications are changing the match options from a continuous colored ring to four discrete choices (alternative forced choice, AFC) on each trial and providing the animals with feedback after each trial. Could these modifications impact the interpretation of the results? We do not believe so. First, we collected data in human subjects on the AFC version of the task, and despite the limitations of these data, the pattern of results is consistent with data collected in humans on the continuous colored-ring version (*SI Appendix*, Fig. S2; the data are limited because they were collected using an online platform with insufficient data in individual participants to perform individual quantitative analysis). Second, we see no evidence that providing feedback reduces errors in matches. On the contrary, the data collected in monkeys (using feedback) show pronounced biases.

Additional data in humans on an AFC version of the task in which feedback is provided after each trial, with sufficient data in individual subjects for quantitative tests, will be useful. But the present evidence, taken together, makes it unlikely that those data will not show cognitive biases, for several reasons. First, the AFC-task (with feedback) deployed in monkeys shows clear evidence for cognitive biases in individual animals, so the task is sufficient to recover cognitive biases should they exist. Second, the mixture-model analysis of data collected in human subjects shows evidence for 4 to 5 attractor points in both the continuous-ring version of the task and the AFC version of the task (*SI Appendix*, Fig. S2). The larger number of attractor points in humans compared to monkeys must be explained by differences in postreceptoral processing, i.e., some form of cognitive difference. Third, feedback is not sufficient to eliminate biases in the AFC task, as clearly evident in the monkey data.

## Toward a Perceptually Uniform Color Space Unconfounded by Language.

Ample behavioral and neurophysiological data support the hypothesis that the perceptual systems that enable color vision are similar in macaques and humans (34, 36, 37, 49). The weight of this prior evidence gives us confidence that the present results obtained in monkeys also apply to humans. That is, it seems likely that the CIELUV color space is not perceptually uniform for human observers tested on the present task, a hypothesis we confirm (Fig. 6 and *SI Appendix*, Fig. S2) and which comports with other findings (42, 46, 47). The nonuniformities in CIELUV and other nominally uniform colorspaces likely have multiple sources. Colorspaces are generally constrained by a small number of defining parameters and are designed for specific viewing conditions and paradigms, which may partly explain why achieving a truly uniform color space has not been possible. But a truly perceptually uniform color space, defined by equal separations across a large set of tasks that tap both small and large color differences, may simply be unachievable, not for technical reasons but because the concept does not align with how the brain uses color (50). So, we sought to narrow the goal, to achieve a perceptually uniform color space for the present task.

The analysis of datasets collected in humans shows evidence for both cognitive biases and stimulus-space nonuniformities (Fig. 6 and *SI Appendix*, Fig. S2), but teasing apart the relative contribution of these mechanisms is challenging. The aggregate behavioral data in macaques are useful, therefore, because they do not show evidence for consensus cognitive biases and are explained entirely by stimulus space nonuniformities, providing an opportunity to reconstruct a uniform color space.

**Fig. 7.**
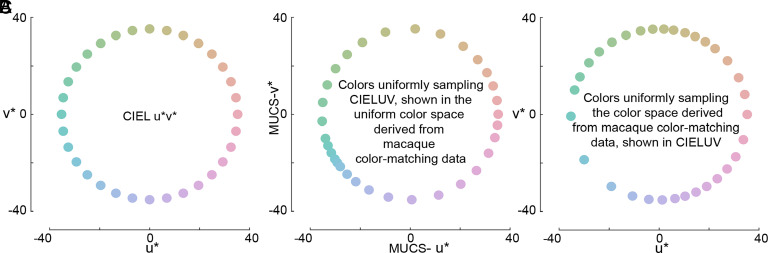
Perceptually uniform color space derived from the color-matching data in macaque monkeys. (*A*) CIELUV color space with 64 color samples at even intervals in hue angle. (*B*) The same color samples plotted in the uniform color space derived from macaque monkeys; note that the axes are not CIELUV but MUCS (macaque uniform color space). (*C*) Colors sampled evenly from the uniform color space derived from macaque monkeys, projected into CIELUV.

To generate such a space, we computed, empirically, a transformed color space such that the macaques would, on average, show no choice bias. We refer to this space as the MUCS. When colors evenly sampled from the CIELUV space (Fig. 7*A*) are plotted within this macaque-derived uniform color space, colors are bunched around the teal part of the space, and to a lesser extent, around the peach-colored part of the space (Fig. 7*B*). We can also take colors sampled at uniform intervals in MUCS and project them into CIELUV (Fig. 7*C*). This arrangement shows relative bunching around lime colors and lavender colors, which correspond to the colors of the poles of the S-cone-opponent axes. These results provide clues to the origin of the stimulus space nonuniformity in CIELUV. Future work could extend the approach to stimuli that sample more of the gamut of human color perception and other tasks.

## Discussion

Color categories are assumed to bias results in simple color-matching tasks and can be modeled as attractors, repellers, or a combination (43). Here, we leverage this assumption to test whether monkeys have color categories. Monkeys performing a color-matching task showed consistent biases in errors across individuals, like humans. But compared to humans, monkeys showed fewer regions of color space with consistent biases, and the biases were explained simply by nonuniformities in the color space. Biases in humans, meanwhile, pointed to cognitive explanations as well as stimulus space nonuniformities. The results imply that humans show consensus color categories and monkeys do not.

The present results are consistent with the idea that color ordering is innate even if consensus color categories are not. This conclusion stems from the similarity matrices, which show structure along the inverse diagonal. Such structure would not be evident if the monkeys possessed no capacity for color ordering. Moreover, one animal showed clear evidence of a private color category, indicating that monkeys possess the capacity to form color categories, even if they show no consensus color categories. Contrary to longstanding arguments in empirical philosophy (51), the present results imply that perception by itself is not sufficient to generate consensus color categories. The present results comport with a growing body of work showing that color perception is not necessary for the acquisition of rich conceptual knowledge about color (26, 52).

A salient difference between monkeys and humans is the expression of language, which provides a plausible mechanism by which an innate potential to form color categories could achieve consensus. Color categories reflect the behavioral relevance of colors in the world (23, 53, 54), so color categories must, at some level, be adaptable, i.e., not constrained by innate hard-wiring. Moreover, behavioral relevance, at least for humans, is partially culturally determined, introducing a role for language in shaping consensus color categories. Taken together, prior work demonstrates that consensus color categories can be attained without appeal to innate mechanisms (23–26); and the present work shows that innate similarities between monkeys and humans are not sufficient to produce consensus color categories.

But are there possible nonlinguistic explanations for the difference in results obtained in monkeys compared to humans? Monkey brains are considerably different from human brains in many respects, and there may be unrecognized physiological differences that account for the behavioral differences. Yet monkeys have similar cones, similar color-detection thresholds (36, 55), similar visual cortex extending through the ventral visual pathway (49, 56), and they are thought to show similar visual cognition to humans, all of which suggests that the differences between monkeys and humans described presently cannot be explained by differences in the normal operation of the visual system. This leaves differences in language and cognition.

Could macaques be born with innate color categories, but lose them? This seems unlikely. The animals studied presently live in social colonies with enrichment, including natural fruits and colored toys. Such an environment is not one of deprivation and provides sufficient experience for the expression of all other innate behaviors that have been assessed, including vocalizations, foraging, face recognition, grooming, and sexual function.

What explains the stimulus space nonuniformities? The mixture-model results in macaque monkeys are strikingly different from those in humans but are similar in one regard: they both show repeller points aligned with the poles of the S-cone axis (compare the zero-crossings of the positive slopes in Fig. 1*C* with those in Fig. 2*C*; *SI Appendix*, Fig. S2) (15, 38, 43). This commonality suggests that the contribution of S-cone signals to suprathreshold color matching may not be accurately reflected in CIELUV, introducing nonuniformity in the color space even though it is often assumed to be perceptually uniform. Such an inaccuracy may reflect differences between threshold measurements versus appearance measurements, and corresponding differences in circuits handling S versus L/M signals (57, 58).

It might be assumed that biases of cognitive and stimulus-space origin are independent. But it is plausible, indeed we think likely, that these two mechanisms interact. For example, a stimulus-space nonuniformity could reflect a cognitive mechanism employed to adapt color perception to the task at hand. The pattern of results in the only dataset with robust individual-level data (43) (*SI Appendix*, Fig. S3) suggests that all apparent consensus categories manifest in the mixture-model analysis of the population data engage a combination of the two mechanisms (Fig. 6). Future work aimed at developing models that combine both mechanisms will be necessary to address the question decisively.

Finally, the pattern of biases observed across three independent datasets collected in English-speaking participants supports the existence of 4 or 5 consensus color categories (*SI Appendix*, Fig. S2). How should we reconcile this number with the larger number of color categories recovered in many studies of color categories? We hypothesize that the color categories recovered in the matching task reflect not simply the existence of a color category but also other factors such as communicative efficiency and the extent to which the category is useful. The categories recovered in matching tasks appear to correspond to regions in color space with significantly high color-naming concordance across cultures (6), suggesting commonality across human populations in terms of which color categories are useful and thus named. It will be interesting to test whether the number of consensus color categories in a given population (of humans or monkeys) can be altered by introducing a change in consensus behavioral relevance.

## Methods

### Subjects.

Data were collected in four adult male rhesus macaques (*Macaca mulatta*) (“PO, CA, BU, and MO”) weighing 8 to 10 kg. All experimental procedures were approved by the Animal Care and Use Committee of the National Eye Institute and complied with the regulations of the NIH. Plastic headposts were mounted with sterile surgical procedures, using procedures described in detail elsewhere (59). The animals were acclimatized with positive reinforcement to sit in a custom-made chair positioned with the eyes 70 cm in front of a computer monitor and to perform visual tasks as described below. At the beginning of each testing session, we positioned a mouthpiece to deliver fluid reward to the animal. An infrared camera was directed at the eye to monitor eye position with the ISCAN system. The precision of the eye tracking was ∼0.3°.

### Behavioral Task.

The animals were trained to perform a 4-Alternative Forced Choice (4-AFC), Delayed Match to Sample task, in which they were shown a colored cue and rewarded for selecting the match option that had the identical color (Fig. 1). The colors on each trial were drawn from a set of 64 colors that evenly sample hue angle of CIELUV color space (42). Each trial was initiated when the animal fixated a small cross at the center of the screen; trials were aborted if the animal did not maintain fixation until the fixation cross disappeared toward the end of the trial. Fixation was defined as within a 1.5° wide area centered on the fixation cross, well within the precision of the eye tracker. The trial sequence was as follows. 50 ms after initiating the trial by fixating the central cross, a 3°-diameter “cue” of a color randomly drawn from the set of 64 colors appeared for 750 ms. The cue was positioned on the monitor at the same location for all trials during a given daily recording session. From day to day, the position of the cue could vary, from 2.5° to 6° eccentricity (measured at the center of the cue) and be at any angle from fixation. The cue was followed by a gray screen for a brief “memory” period of 600 to 900 ms, after which four match options appeared along an arc at an eccentricity of 6°.

One match option was always a direct match to the cue, and the other three were randomly sampled without replacement from the remaining 63 stimuli. The match options had the same shape and size as the cue (3°-diameter discs); they were evenly spaced along the arc, with a gap of 2° separating each option. In all animals except one (CA), the arc along which the match options were placed was in the visual hemifield opposite to the cue. The exact position of the arc within the hemifield varied from trial to trial, so the animals could not anticipate where the choice options would appear. Animal CA had a small scotoma spanning ∼3° of visual angle in a quarter of the visual field as the result of a ∼3 mm diameter V1 lesion, so the cue and choices were placed in the same (intact) hemifield, taking care to avoid overlap of the position of the cue and the choices.

After a random period from 500 to 1,000 ms, the fixation cross disappeared, instructing the monkey to direct its eyes to one of the choices. This random period helped guard against the animals making impulsive responses. Reward was given only if the animal selected with an eye movement the choice that was identical to the cue. If the monkey failed to make a choice within 5 s or broke fixation at any point before the termination of the fixation cross, the trial was aborted.

The experiment was controlled with custom software written in MATLAB and Psychtoolbox (60).

#### Stimuli.

Stimuli were 3° diameter discs presented on a Cambridge Research Systems Display++ screen. Colors were defined to be on an equiluminant plane in CIELUV color space, with the luminance matched to the adapting gray background (L* = 76.0693, 38.5 cd/m^2^; adapting field chromaticity was xy_1931_: 0.2684, 0.2409). The stimulus set included 64 colors, evenly sampling CIELUV hue angle (5.625° between adjacent stimuli), of equal CIELUV saturation (radius 37), the highest saturation possible for a set of stimuli of equal saturation and luminance given the gamut of the display. Luminance contrast noise was randomly added to each pixel of the cue and the match options. The luminance added to each pixel was updated every frame and drawn uniformly from a continuous range of ±5 L* units (the resulting stimulus looks like a colored disc viewed behind a thin veil of television snow). CIELUV was used to define the stimuli because it has an associated chromaticity diagram, but the stimuli can readily be transformed into other spaces such as CIELAB and DKL.

The color stimuli corresponding to the poles of the cone opponent cardinal axes, labeled in the polar mixture model plots, were computed as follows. CIELAB and CIELUV values were transformed to XYZ coordinates using the PsychToolbox functions “LabToXYZ” and “LuvToXYZ,” respectively. An XYZToLMS matrix was constructed using MATLAB’s “mldivide” function with the Smith-Pokorny cone fundamentals and the CIE XYZ standard observer color-matching functions as inputs. The PsychToolbox function “ComputeDKL_M” was then used to compute a conversion matrix for converting between LMS and cone opponent cardinal axes that define the DKL colorspace. The code which accomplishes the above is available here (62).

#### Human data.

Data from published reports using two related tasks completed in human subjects were kindly provided by Bae et al. (43) and Timothy Buschman (38). Both these datasets involved a paradigm in which participants matched the color of a cue to a ring of colors showing a continuous progression of colors around the color circle (a “color wheel”). The results of the two prior studies are consistent with each other: when analyzed with a mixture model, both datasets show four color categories corresponding to blue, green, orange, and pink (*SI Appendix*, Fig. S2); moreover, the results on a version of the task that omits the memory delay period also recover these four color categories (43). This prior work shows that the results of the color-matching task are reproducible and robust. It is therefore likely that the results of the present version of the task, which is distinguished from the prior work by providing as options discrete targets as opposed to a continuous colored wheel, would also be similar.

But to test this likelihood, we recruited human participants via Amazon Mechanical Turk to perform the same task used in the macaque monkeys. All human participants provided informed consent and were compensated financially for their participation. The procedures were approved by the Institutional Review Board of the NIH Intramural Research Program. To request a trial, participants used a mouse to adjust the location of a cursor to click on a fixation cross, after which a cue was shown to one side of the fixation cross, and the cursor disappeared. The cue was displayed for 750 ms. After the cue was extinguished, a fixation cross was shown but the cursor remained hidden to deincentivize mouse movement (1,500 ms). Four choices were then shown, and the cursor reappeared; participants made their selection by using the mouse to move the cursor to their choice and clicking the mouse. Most other aspects of the experimental design were the same as the experiment deployed with the monkeys: 64 colors of equal saturation and luminance (we assumed the monitor that each online participant was using matched the sRGB standard), evenly sampling CIELUV. We did not provide feedback to human participants [in line with prior human work (38, 43)].

We collected data from 72 participants in blocks of 400 trials; 400 to 2,000 trials from each (mean: 639 trials).

The pattern of results was consistent with the published studies using the continuous ring of colors, recovering four significant color categories corresponding to blue, green, orange, and pink (*SI Appendix*, Fig. S1).

### Data Analysis.

#### Psychometric functions.

Psychometric functions (Fig. 2*A* and *SI Appendix*, Fig. S1) were estimated with a Weibull cumulative density function:

[1] $$\begin{array}{c} y=\zeta +\left(100-\zeta -\gamma \right)*\left(1-e^{-{\left(\omega /\lambda \right)}^{k}}\right), \end{array}$$

where animal performance (y) is a function of trial difficulty (*ω*), computed for each trial as the angular difference between the color of the cue and the color of the foil with the closest color to the cue. The other parameters are the floor of the function (*ζ*), the ceiling (*γ*), slope (*λ*), and inflection point (*k*). All completed trials were included in the analysis of the psychometric functions. The number of completed trials for each animal were 76,121 (PO); 54,555 (CA); 24,526 (BU); 54,252 (MO).

#### Mixture modeling.

Following experiments in human subjects, we analyzed the distribution of color matches made to each color using a mixture model (43, 45); this model assumes that the shape of the distribution is normal and provides an estimate of the width of the distribution, the offset (or bias) of its peak relative to the target color, and the guess rate. Prior work has done this analysis with a von Mises distribution (43, 45). We found it simpler to implement it with a Gaussian function, f(θ):

[2] $$\begin{array}{c} f\left(\theta \right)=\alpha \cdot e^{-\frac{{\left(\theta -\mu \right)}^{2}}{2\sigma^{2}}}+\zeta , \end{array}$$

where *α* is the height of the curve’s peak, *θ* is the hue angle, *σ* is the SD (width), *μ* is the center of the peak (so the offset is *θ*-*μ*), and *ζ* is the floor (guess rate). To do the analysis, we used the MATLAB fit function, which was provided with the number of times each choice color was an option for the given cue across all completed trials and produced the best-fitting function along with the 95% CI values for those parameters (*SI Appendix*, Fig. S4; note that the tails of the distributions in *SI Appendix*, Fig. S4 reach an asymptotic floor, justifying the use of the simpler Gaussian fitting procedure). The offset values for each cue were smoothed with a moving average spanning three colors (16.875°) (Figs. 2 *B* and *C* and 5*A* and *SI Appendix*, Fig. S5). Negative-slope zero-crossings in which the 95% CI exceeded the zero-crossing were considered category centers (Fig. 1).

The width of distribution of matches varied among the colors; this was also observed by Bae et al. (43) (see their Fig. 7). This variation implies that the assumption of uniformity of the colorspace is not valid. Moreover, such a nonuniformity could yield an offset in the mixture model (Fig. 3), so any offsets recovered by the mixture model need not require a cognitive origin. The alternative hypotheses for the origin of offsets recovered by the mixture model (a cognitive origin versus a stimulus-space nonuniformity), prompted us to analyze the data with a more sensitive model, the Target Confusability Competition model (48). We recognize that in principle the mixture model could distinguish these alternative hypotheses, but we encountered some limitations using the mixture model that were readily overcome by the TCC model.

#### Modified target confusability competition model (TCC-v).

The key elements of the TCC model are a similarity function, which determines the similarity between stimulus *s*_*i*_ and stimulus *s*_*j*_ through a nonlinear mapping of distance in colorspace to perceptual similarity, and a value of $d^{\prime }$, which can be thought of as describing the amount of noise acting in the system. These two elements can be used to predict the probability that a choice of color *s*_*j*_ will be picked from the set of $\left[s_{j1}...s_{\mathrm{jn}}\right]$, on a trial where the cue is *s*_*i*_.

The probability of a particular choice being selected on a particular trial ($p_{t}\left(\;\text{selected choice}=\;{\text{choice}}_{n}\right)$) is dependent on the cue, the set of choices, the similarity function (f(θ)), the value of $d^{\prime }$, and the perceptual distances between stimuli (*D*).

[3] $$\begin{array}{c} p_{t}\left(\;\text{selected choice}=\;{\text{choice}}_{n}|\;\text{cue},\;{\text{choices}}_{i:j},f\left(\theta \right),\delta ,D\right), \end{array}$$

where f(θ) is the Gaussian equation (Eq. **2**), *δ* is the value of $d^{\prime }$, and *D* is the perceptual distances between stimuli.

The TCC model has been deployed with the assumption that the colorspace is perceptually uniform (48); this assumption is implemented as a single similarity function fit for all stimuli. But, as Schurgin et al. (48) demonstrate, the similarity function need not be fixed (see their figure 1D and extended data figure 5). Our implementation of the model, which we call TCC-v, permits the similarity function to vary for each cue (the “v” is for vary).

We created four versions of the TCC-v model: the null model (with only parameters for the Gaussian width of the similarity function and $d^{\prime }$, and thus no allowance for bias), the cognitive bias model (with 64 parameters corresponding to offset values shifting the peak of the similarity function for each stimulus to higher or lower hue angles, in addition to $d^{\prime }$ and Gaussian width), and the stimulus-space nonuniformity model (with 64 parameters corresponding to the relative distance between each pair of neighboring stimuli, in addition to $d^{\prime }$ and Gaussian width). Finally, the free-similarity model, does not presuppose any specific similarity function; every cell in the similarity matrix is an independent parameter. This flexibility allows for patterns of similarity that are not captured by our hypotheses.

In fitting the model parameters, our goal is to minimize the negative log likelihood (NLL) of the observed data. The NLL is computed as the sum of the negated log of the probabilities of the choices that were selected being selected.

[4] $$\begin{array}{c} \;\text{NLL}=\;\text{sum}\left(-\left(\log\left(p_{t}\left(\;\text{selected choice}=\;{\text{choice}}_{x}\right)\right)\right)\right), \end{array}$$

where ${\text{choice}}_{x}$ was the choice that was selected on each trial.

The parameter estimates for the free parameters used by the model are iteratively updated until the model reaches a stable minimum NLL.

The null model is defined by f(θ) (where *α* = 1, *ζ* = 0, *μ* = 0, *σ* is 1 free parameter) and $d^{\prime }$ (free parameter), and assumes *D* to be uniform. The cognitive bias model is defined by f(θ) (*α* = 1, *ζ* = 0, *μ* is 64 free parameters, *σ* is 1 free parameter) and $d^{\prime }$ (free parameter), and also assumes *D* to be uniform. The stimulus-space nonuniformity model is defined by f(θ) (where *α* = 1, *ζ* = 0, *μ* = 0, *σ* is 1 free parameter) and $d^{\prime }$ (free parameter), but specifies for each pair of neighboring stimuli a unique value for *D* (64 free parameters). Note that the cognitive bias model and the stimulus-space nonuniformity have the same total number of free parameters (66). The free similarity model is not defined by f(θ); it is defined by $d^{\prime }$ and the similarity matrix. The similarity matrix is defined by 4,096 (64^2^) parameters, one for each combination of cue and possible choice (note that the free similarity matrix is not required to be symmetric). In practice, when fitting a free similarity model we fix $d^{\prime }$ rather than allowing it to be a free parameter.

$p_{t}\left(\;\text{selected choice}=\;{\text{choice}}_{n}\right)$ is the probability that a sample drawn from an independent normal distribution $\left(X_{i}∼N\right)$ is the highest of such samples drawn for all the choices (*i*: *j*) on a particular trial. The mean (*m*) of each distribution is defined by the similarity value for that cue/choice combination, multiplied by $d^{\prime }$, and has a variance of 1.

[5] $$\begin{array}{cc} p\left(\;\text{selected choice}=\;{\text{choice}}_{n}\right) & =p\left(X_{i}∼N\left(m_{n}\cdot \delta ,1\right)\right.\\ & >\;\max\left.\left(X_{i+1:n}∼N\left(m_{n}\cdot \delta ,1\right)\right)\right), \end{array}$$

where $m_{i:j}=f\left(|\;{\text{cue}}_{i}-\;{\text{choices}}_{i:j}|\right)$, and *δ* is the value of $d^{\prime }$.

We used an AFC paradigm as opposed to a continuous response space, so we can take advantage of an alternative computational method for estimating $p_{t}\left(\;\text{selected choice}=\;{\text{choice}}_{n}\right)$, using correction factors provided by McGraw and Wong (61) (their table 3). This decreases the amount of time taken to fit the TCC-v models. The method for computing $p_{t}\left(\;\text{selected choice}=\;{\text{choice}}_{n}\right)$ in the original TCC model of Schurgin et al. is provided here: modelPDF in TCC_Code_InManuscriptOrder Model TCCUncorrelated.m from https://osf.io/j2h65/. The method we used is provided here: https://github.com/NEI-LSR/TCC_AFC.

When fitting the free similarity matrix model, we fix $d^{\prime }$ to provide a constraint on the floor and ceiling of values in the similarity matrix. $d^{\prime }$ is strongly correlated with the range of the values in the similarity matrix; the range of similarity values in the similarity matrix has a maximum span of 0 to 1. Equivalent NLL values can be obtained either by restricting the range (e.g., 0.49 to 0.51) and a high value of $d^{\prime }$ (e.g., 20), or by having a larger range (e.g., the full 0 to 1) and a low value of $d^{\prime }$ (e.g., 0.1). If $d^{\prime }$ is not fixed, the model is as likely to assume a restricted range as it is a larger range (though still bounded between 0 and 1) but will take a long time to converge. Fixing $d^{\prime }$ impacts the specific values (akin to increasing or decreasing the contrast of the similarity matrix image) but it does not impact the interpretation of the similarity matrix. We chose a $d^{\prime }$ value of 1 which is a reasonable estimate for our task (48).

In the original TCC model, the similarity function uses two parameters that define a Gaussian function representing perceptual noise and an exponential function; these two functions are convolved (see figure 1F of ref. 48). We simplify the similarity function such that it is defined by a Gaussian alone. The cost of this is that it does not allow for a distinction between the impact of perceptual noise (where the curve flattens off approaching perceptual distances of 0) and similarity (the general shape of the function). In practice, we found that these parameters were highly correlated, and that reduction to a single parameter substantially reduced the computational cost of model fitting and produced parameter estimates that were more resistant to variation in model-fitting starting values. This simplification also made it easier to modify the model; for example, to allow for the peak of the function to not be at 0 (this affords the TCC-v model the same ability as the mixture model to capture offsets).

#### Quantitative model comparison.

To compare the relative performance of models, we computed BIC values for each model from the NLL. The BIC values provide a unit by which we can assess whether the differences between NLL are meaningful, penalizing for number of parameters and number of trials. BIC also allows us to compare models with differing numbers of parameters (though note that the cognitive bias and stimulus-space nonuniformity models have the same number of parameters).

[6] $$\begin{array}{c} \;\text{BIC}=k\ln\left(n\right)-2\ln\left(\hat{L}\right), \end{array}$$

where $\hat{L}$ is the maximized likelihood of the model, *n* is the number of trials, and *k* is the number of parameters.

To compare the relative performance of the cognitive bias and stimulus-space nonuniformity models, we performed 100 bootstrap iterations of the analysis. Each bootstrap drew 24,526 trials from the total number of completed trials for each animal (24,526 was the minimum number of completed trials among the four animals). Both models were then fit to each bootstrap iteration, and the BIC values were computed (Fig. 4*D*).

#### Reverse-engineering a uniform color space from the macaque color-matching data.

The present results imply that CIELUV is perceptually nonuniform; that is, that it samples with variable density the true underlying perceptual colorspace. The parameters of the stimulus-space nonuniformity model describe the relative positions of the stimuli, as determined empirically. After converting from interneighbor relative distances to polar angles, these can be thought of as the hue angles for the stimuli that we used, represented now in a behaviorally derived color space which we refer to as the Macaque Uniform Color Space (MUCS) (Fig. 7*B*). The inverse transformation is also possible; we can define a set of hue angles that are uniformly distributed in MUCS and convert them into CIELUV. For example, if we define hue angle *i* in MUCS, we can reparameterize that hue angle such that it is defined by its angle relative to the two experimental stimuli on either side of it (hue angle *i* is at y% of the angular distance on the path between experimental stimuli *a* and *b*). We can then plot MUCS hue angle *i* in CIELUV by plotting it at the location which is y% along the path between experimental stimuli *a* and *b* in CIELUV. Such a set of hue angles is shown in Fig. 7*C*.

The MATLAB script MUCS.m (62) will generate a user-defined number of colors sampled evenly from the behaviorally generated color space.

## Supplementary Material

Appendix 01 (PDF)

## Data Availability

The data and analysis pipeline are available on Github (63). A self-contained folder with all the resources for the manuscript is also provided on Zenodo (62).
