# Supplementary material for: The origin of color categories
Source: Proc Natl Acad Sci U S A. 2024 Dec 30;122(1):e2400273121. doi: 10.1073/pnas.2400273121 (PMC11725794; doi:10.1073/pnas.2400273121)
Supplement: Supplementary file 1 — Appendix 01 (PDF) [file pnas.2400273121.sapp.pdf]

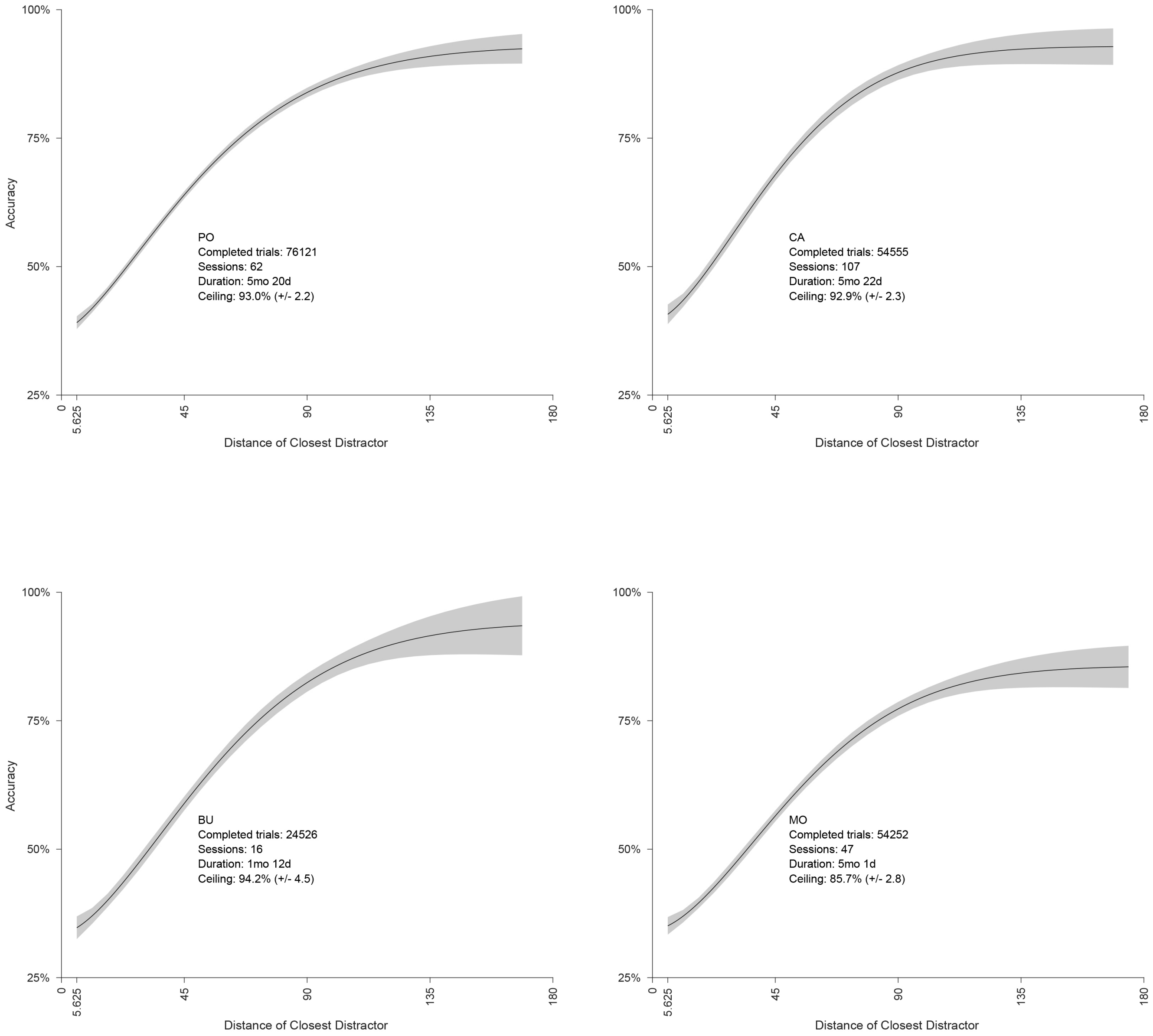


**Figure S1. Psychometric functions for the four individual animals (PO, CA, BU, MO) on the color-matching task illustrated in Figure 1.** Completed trials for the four animals were: 76121; 54555; 24526; 54252.

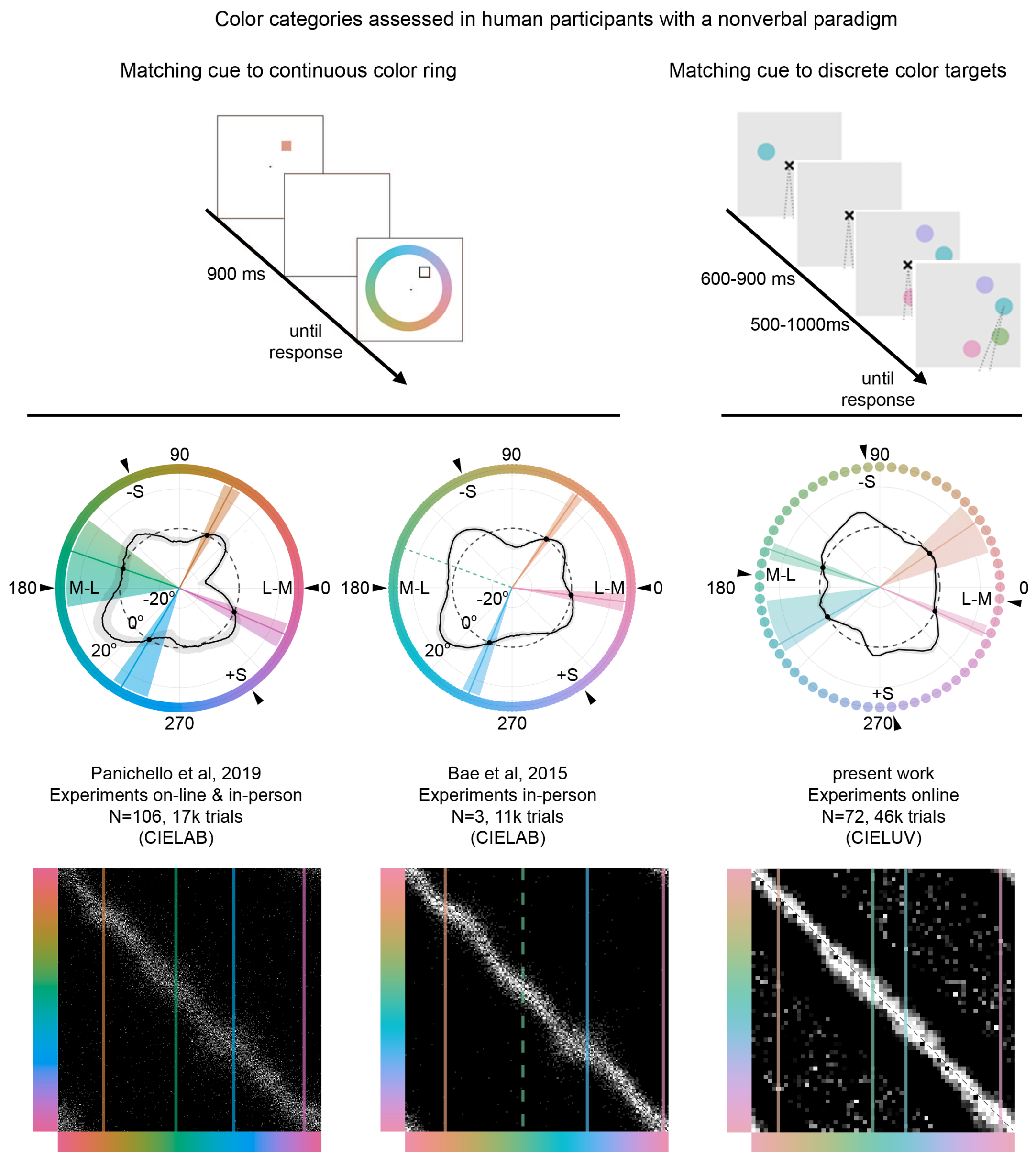


**Figure S2. Color categories assessed in human participants with a nonverbal paradigm.** Prior work by Panichello et al. [1] and Bae et al. [2] has used a task in which participants match the color of a cue to a continuous ring of colors; those published results were obtained with a combination of in-person experiments and on-line experiment, with a memory delay and without. The results are broadly consistent, recovering four color categories by mixture-model analysis, corresponding to blue, green, orange and pink. The present work adapted the paradigm so that the match options in each trial were four discrete targets randomly drawn from 64 colors sampling the color space. The data shown on the right of this figure were obtained in human participants, with experiments conducted online with Amazon Mechanical Turk. These results are again broadly consistent with the prior work using the continuous-matching ring in recovering, by mixture analysis, four significant categories corresponding to blue, green, orange, and pink. The bottom row of this figure shows the choice probability matrices for the Panichello et al. [1] and Bae et al. [2] data, and the similarity matrix for our human data, with the mixture-model-identified attractor points highlighted. The stimuli in the prior work [1, 2] were defined in CIELAB, while the stimuli in the present work were defined in CIELUV; this difference in color space is associated with a slight clockwise rotation of the cone-opponent axes. The S axis poles provide a useful landmark; they are associated with positive slopes in all three data sets.

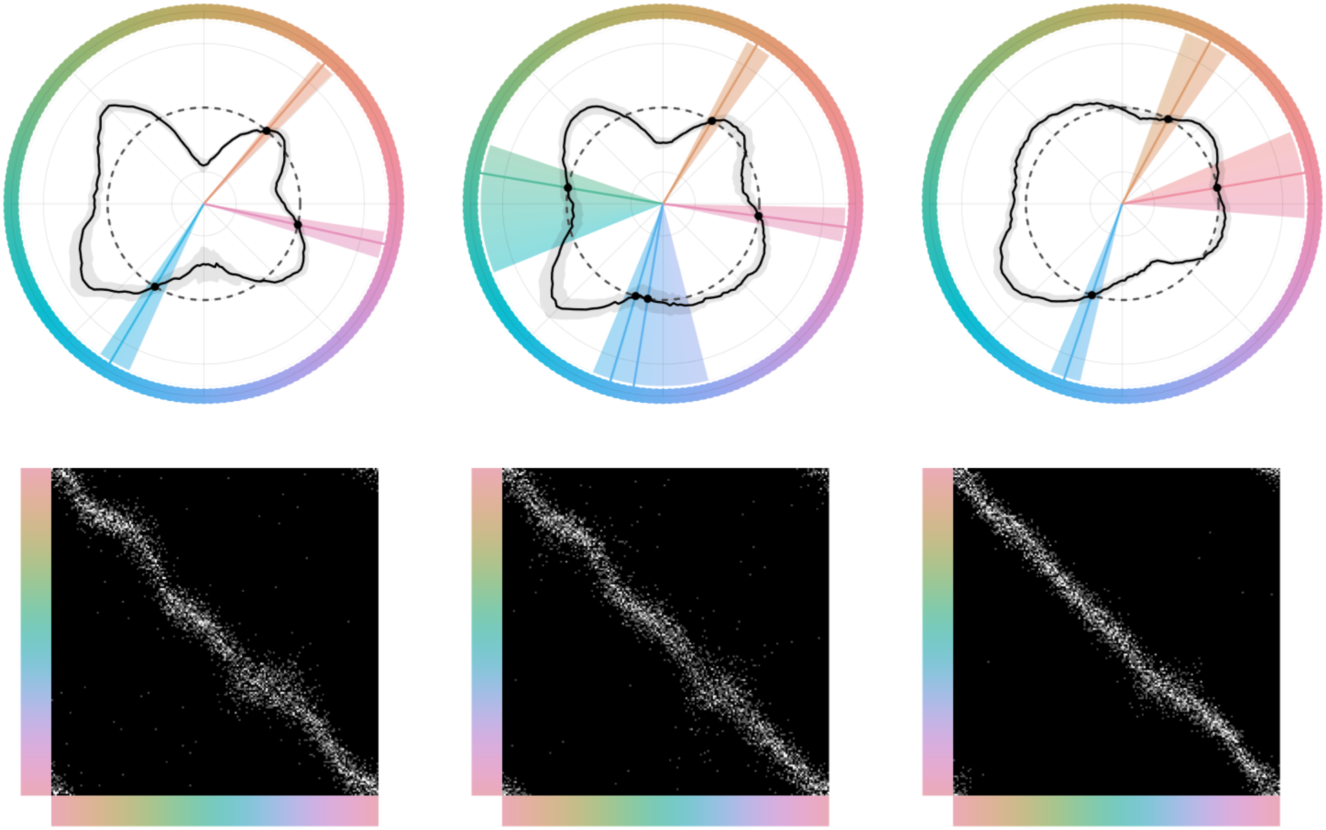

**Figure S3. Models fit to the individual data of Bae et al. [2] (delayed).** The delayed data of [2] consists of data from 3 participants, who completed 3600 trials each, providing sufficient data for a meaningful individual-level analysis. We present individual-level mixture models and choice probability matrices. The third observer (right) appears to have somewhat diminished cognitive biases compared to the other observers. Prior work (such as [2]) has focused on the analysis of aggregated data, partly because this suits the hypotheses they (and we) have sought to test, but also due to technical limitations - using current methods a large amount of data is required, and collecting smaller amounts of data from individuals and then combining it is often a more palatable solution than collecting vast amounts of data from individuals. Future work which considers individual differences will be valuable, in part because it is an open question how consensus terms are created within a culture. For example, cognitive biases might only emerge at the group level as a combination of the biases of many individuals, in the same way that a rich representation of color categories is evident in a distributed fashion across human populations that appear to have relatively low informative color-naming systems [3, 4].

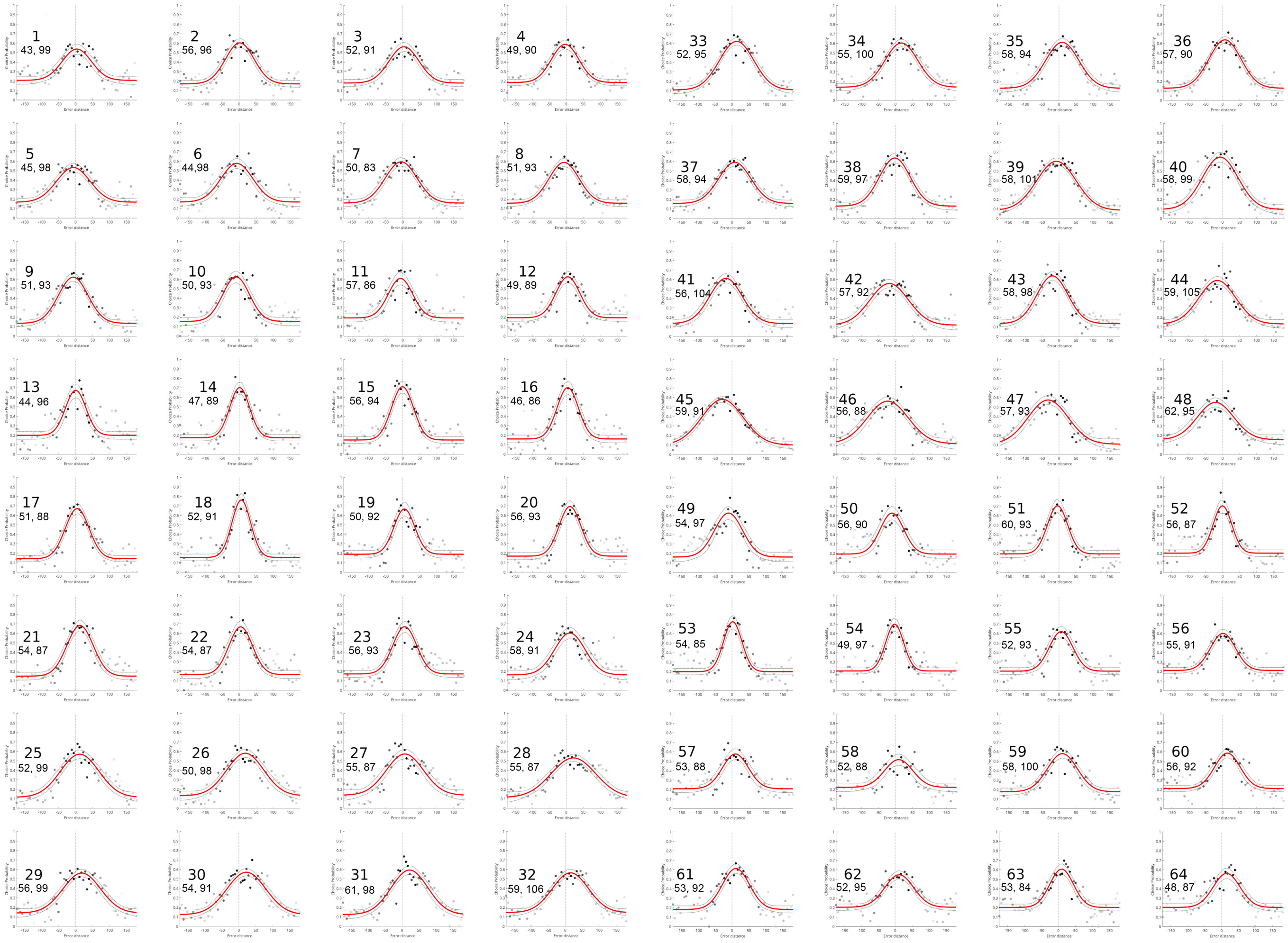


**Figure S4. Gaussian fits from the Mixture Model.** Each trace shows the Gaussian fit for one of the 64 target colors (large number in each panel corresponds to the cue color) used in the color-matching task, as per Equation 1; data averaged over four animals. The extent to which each data point is black versus white indicates the number of trials that provided that choice option, normalized for each cue (the pair of smaller numbers below the cue-color number provides the range: the larger number corresponds to the black symbol, and the smaller number to white); see Methods for how the curves were fit to the data.

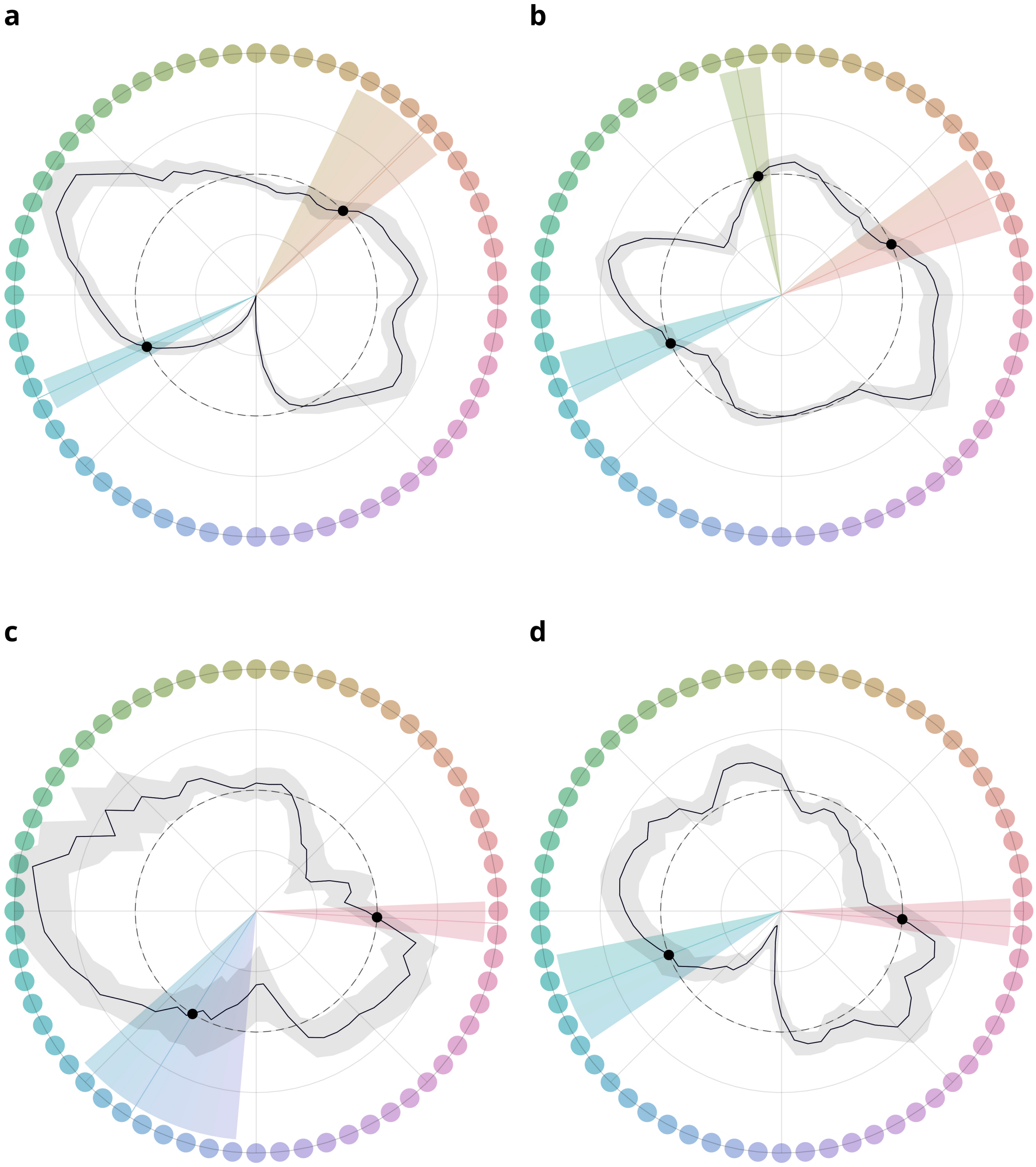


**Figure S5. Mixture-model analysis of the individual data for the four animals in the color-matching task.** Data shown in the same format as Figure 2c. All data for each animal is included (in contrast to Figures 2b,c where data were subsampled to ensure equal contributions from each animal).

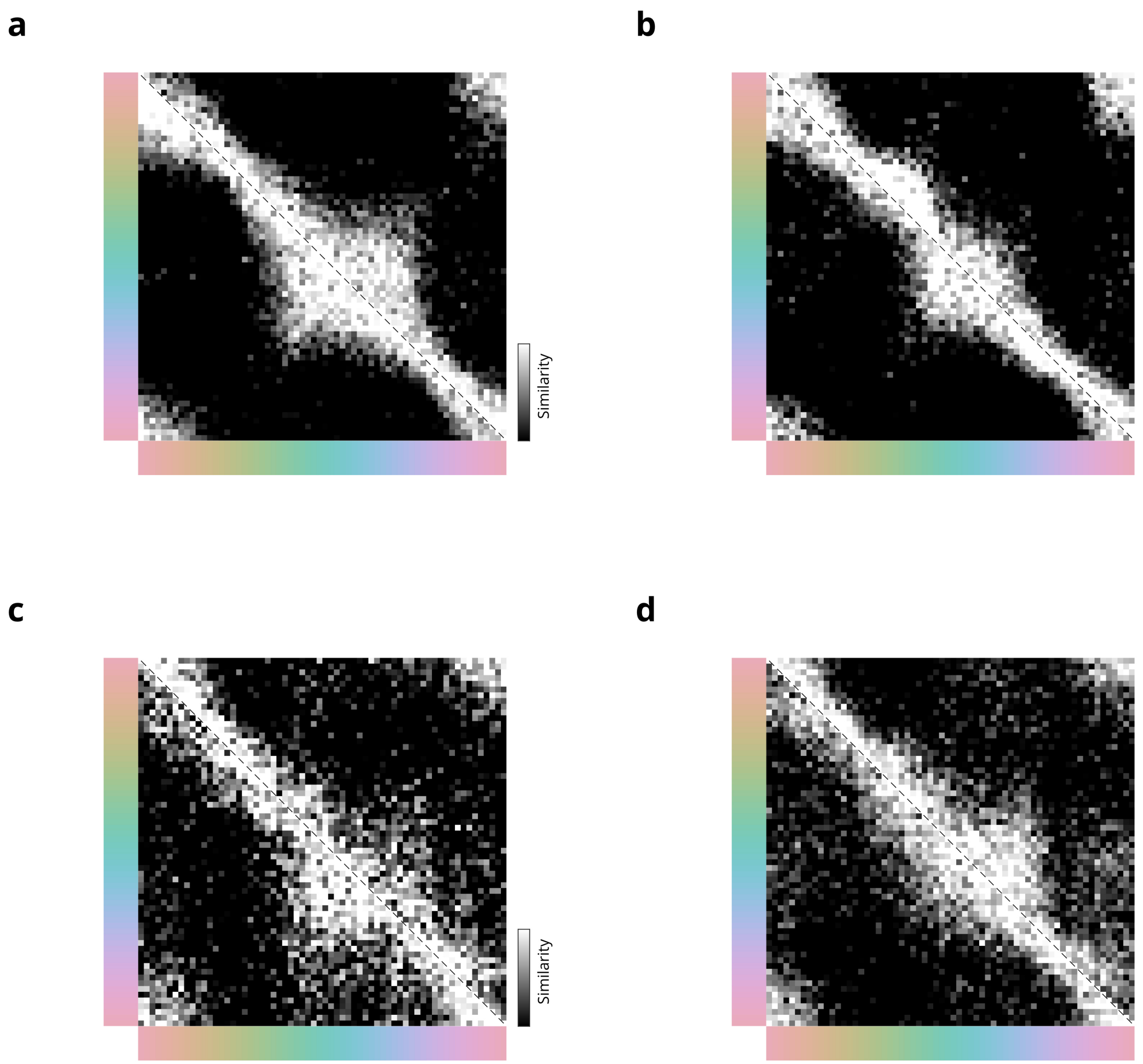


**Figure S6. Similarity matrices for free similarity matrix models, fit to individual data.** The order of the plots is the same as the order in other figures, PO, CA, BU, MO. These plots use the full set of data for each animal. See Figure 3a,b for data averaged across animals, with data subsampled to ensure the same amount of data per animal.

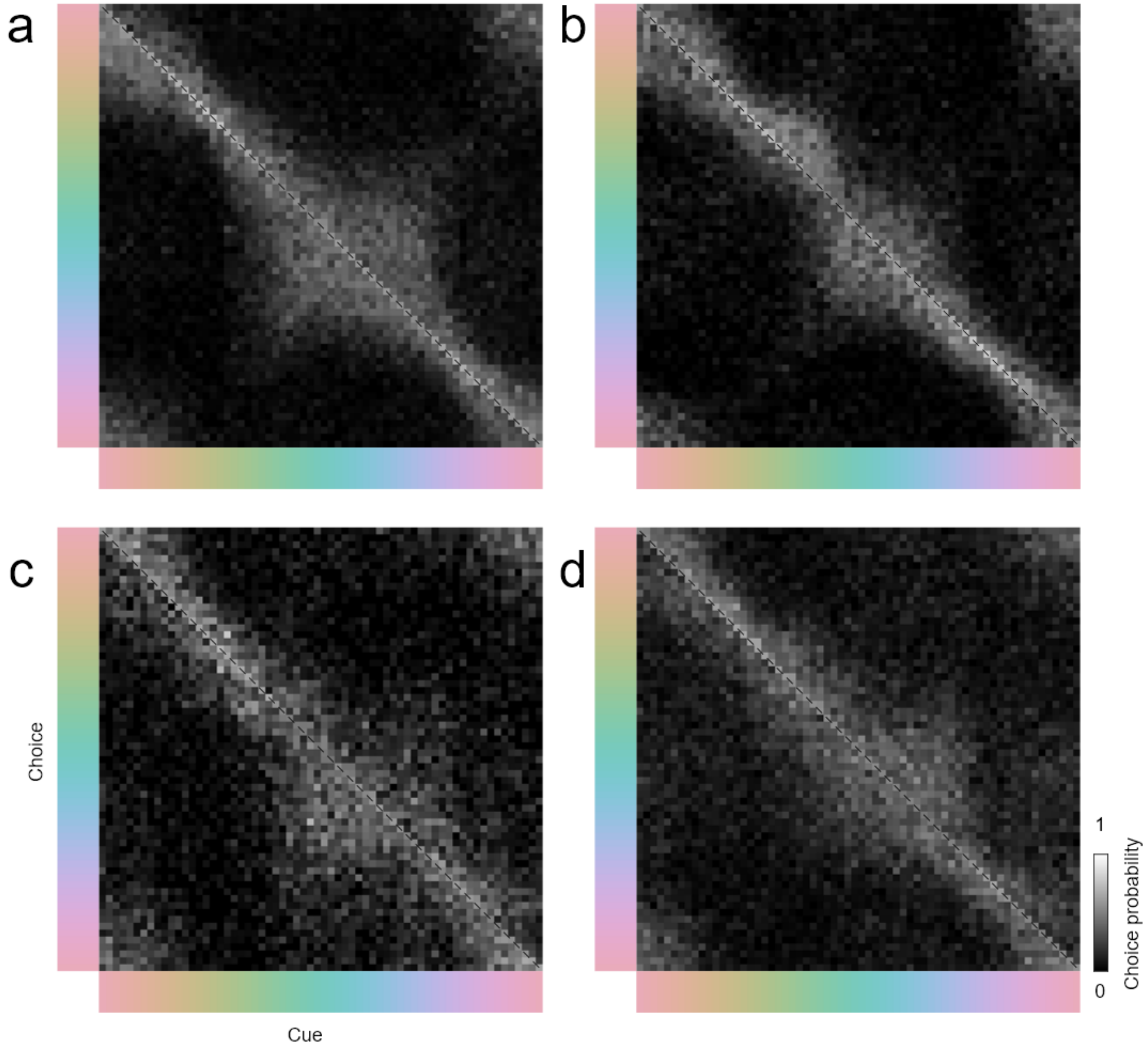


**Figure S7. Choice probability matrices for free similarity matrix models, for the four individuals.** As per the similarity matrices presented, each column represents a cue, and each row represents a choice. Here, however, rather than the similarity between cue/choice pairs we show the probability of selection. Whereas the similarity matrix is the output of model fitting, this matrix is derived more directly from the data: each cell is simply the number of times a particular choice was made divided by the number of times that choice was an option. The identity line, representing correct options, appears artificially inflated (see Methods for further information).

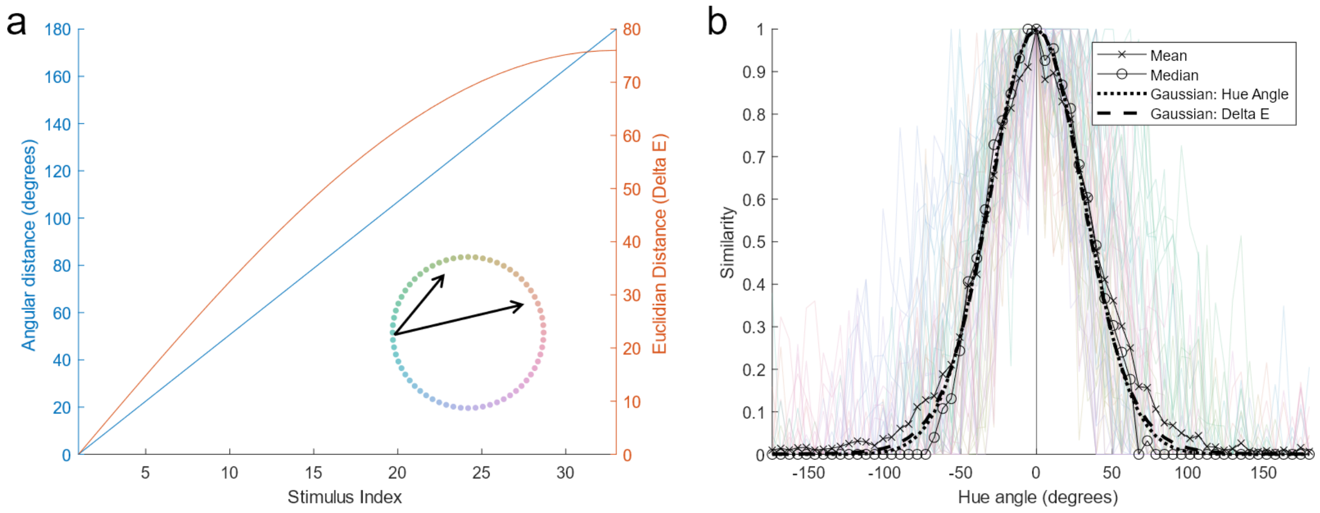


**Figure S8. Comparing distance metrics: angular distance vs Euclidean distance.** We inherit the practice of using angular distance as our distance metric from prior work [1, 2], but another reasonable distance metric exists: the Euclidean distance in CIELAB and CIELUV is referred to as "Delta E". These two metrics are closely correlated at lower values, diverging increasingly at higher values. Using either in our model makes negligible difference, and so in this paper we choose to use angular distance. However, if one were to use a TCC-v model to analyse multi-dimensional data (as opposed to our one-dimensional ring of stimuli) Delta E it seems reasonable that a switch to Delta E would be a sensible path. **a**, Angular distance (hue angle difference) and cartesian distance (Delta E) as a function of stimulus index difference. **b**, Gaussian fits parameterized by each distance metric. Translucent colored lines are the columns from F4a/b, colored by cue. Dotted and dashed lines are Gaussian fits where the Gaussian is computed as a function of hue angle and Delta E respectively. Note how they diverge, but only slightly, and that both align well with the mean and the mode.

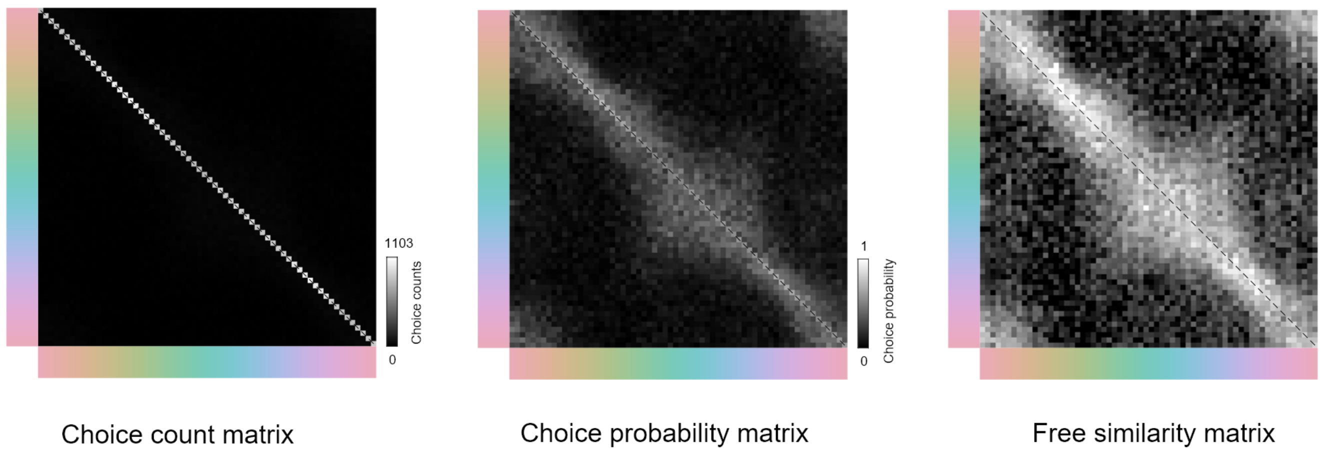


**Figure S9. Comparison of Choice Count Matrices, Choice Probability Matrices, and Similarity Matrices**. Choice count matrices describe the number of times a specific choice is made. Choice probability matrices normalise for the number of times a particular choice was available as a choice option. Similarity matrices are created through model fitting and represent the perceptual similarity between stimuli, and are beneficial since they are not influenced by mixed viability of distractors (see text for further details). Plots shown are for the combined monkey data. For individual data, see SI Figure 5 and SI Figure 6.

## Choice Probability Matrices vs. Similarity Matrices

A choice probability matrix is a graphical representation of the probability that choice $y$ will be chosen given cue $x$ (Si Figure 9, middle). To construct such a matrix, one counts the choices which were made in response to each cue, producing what we can refer to as a choice count matrix (SI Figure 9, left), and then divides each cell by the number of times that each cue/choice combination was presented. This normalizes for the possibility that some cue/choice combinations were presented more than other combinations. This process disregards any information about what the unchosen distractors were on a given trial.

In paradigms which use a continuous response space the set of choices is the same on each trial – the full set of possible choices. This is not the case for our reduced AFC paradigm, and this introduces a potential issue which needs consideration.

Imagine a trial with three distractors which are all close to one another (let's say that they are 5, 6, and 7 colorimetric units away from the cue). On another trial, imagine that one distractor is a much more viable choice option than the other two (let's say the choices are 5, 25 and 26 units away from the cue). The odds of picking the distractor that is 5 units away is different on these two trials - it is more likely on the second trial, because the other distractors are less viable options. This becomes a potential issue because to counterbalance trials such that each cue is shown with the full set of possible distractors would require roughly 2.5 million trials (nchoosek(63,3) * 64). Without counterbalancing, it is not possible to disentangle differences in choice probability which arise due to perceptual similarity (which is what we are aiming to measure) vs differences in distractor viability.

We avoid having to collect a complete set of 2.5 million trials by employing the concept of a similarity matrix. A similarity matrix is the core component of a TCC-v model, and maps the similarity between cues and choices. Using it, the similarity between the cue and each of the choices on a particular trial (and also the similarity between each of the choices themselves) can be used to compute the probability of each choice being selected. While we can compute the choice probability matrix rather simply, as described above, the creation of a similarity matrix requires a model-fitting approach – a similarity matrix is randomly initialized with values, and the likelihood of the data given that matrix is computed, then the matrix is randomly perturbed and the likelihood of the data is recomputed, and then perturbed again in whatever directions seem to increase the likelihood of the data, setting forth an iterative process whereby the similarity matrix is perturbed until a maximum in likelihood is reached, and our best estimate of the underlying similarity matrix is thus settled upon. This is a computationally intensive process, and for datasets with many choices (such as those with a continuous response space), we have been unable to fit similarity matrices. Thankfully, there is a trade-off: data collected with continuous response spaces see less benefit of a similarity matrix over a probability matrix. For such datasets, as the number of trials increases the probability matrix increasingly approximates the similarity matrix. It is expected that choice probability matrices and similarity matrices will be highly correlated and show the same structure.

A related issue, also addressed by employing the concept a similarity matrix, is the artifact caused by the inclusion of a direct match on every trial in our paradigm. This can be thought of

as an unequal counterbalancing which no number of repeated trials would be able to balance out, but it is necessary for our training of the monkeys (since there needs to be a clear rubric for what choices are "correct" and thus rewarded). Note how the choice count matrices and choice probability matrices for our data appear to show an elevated diagonal. The reason this exists in the choice count matrix is clear – those option are simply presented more frequently, but the reason this survives the normalization (by the number of times that each cue/choice combination was presented) is less obvious: any non-match choice which is close to the direct match will always have a viable competitor (the match), whereas the match choice won't always have a viable competitor (since it is possible that the distractors are all distant on a particular trial). This leads to an increased probability of selecting the matching choice relative to an incorrect choice. This issue only affects choice probability matrices; it does not affect similarity matrices.
